# Retrieval of the Clipped Axillary Lymph Node and Its Impact on Treatment Decisions

**DOI:** 10.3390/cancers16173001

**Published:** 2024-08-29

**Authors:** David Detz Jr., Diego Hanssen, Junmin Whiting, Weihong Sun, Brian Czerniecki, Susan Hoover, Nazanin Khakpour, John Kiluk, Christine Laronga, Melissa Mallory, M. Catherine Lee, Laura Kruper

**Affiliations:** 1 Comprehensive Breast Program, Moffitt Cancer Center, Tampa, FL 33612, USA; diego.hanssen@moffitt.org (D.H.); weihong.sun@moffitt.org (W.S.); brian.czerniecki@moffitt.org (B.C.); susan.hoover@moffitt.org (S.H.); nazanin.khakpour@moffitt.org (N.K.); john.kiluk@moffitt.org (J.K.); christine.laronga@moffitt.org (C.L.); melissa.mallory@moffitt.org (M.M.); marie.lee@moffitt.org (M.C.L.); 2 Department of Biostatistics & Bioinformatics, Moffitt Cancer Center, Tampa, FL 33612, USA; junmin.whiting@moffitt.org

**Keywords:** sentinel lymph node biopsy, targeted axillary dissection, pathologic complete response, axillary metastasis, breast cancer, clipped lymph node, neoadjuvant chemotherapy

## Abstract

**Simple Summary:**

Around 30% of breast cancer patients have axillary lymph node metastases present at the time of diagnosis. Historically, axillary lymph node dissection was performed in these patients with significant morbidity associated with the procedure including permanent lymphedema. These patients are now often treated with neoadjuvant chemotherapy to attempt to downstage the axilla and avoid axillary lymph node dissection. To reduce the false negative rate of a sentinel lymph node biopsy, a targeted axillary dissection is often performed to ensure that biopsy-proven metastatic axillary nodes are removed. The aim of our study was to determine how often the clipped node was also a sentinel lymph node, to identify factors that were associated with pathologic complete response following neoadjuvant chemotherapy, and to assess how the clipped node impacted final treatment decisions. These findings aim to help understand and guide surgeons on axillary evaluation after neoadjuvant chemotherapy.

**Abstract:**

We examined clinically node-positive (cN+) breast cancer patients undergoing neoadjuvant chemotherapy and clipped lymph node (CLN) localization to determine the rate of CLN = non-sentinel lymph node (SLN), the factors associated with cN+ to pN0 conversion, and the treatment impact. We conducted a single institution review of cN+ patients receiving NAC from 2016 to 2022 with preoperative CLN localization (N = 81). Demographics, hormone receptor (HR) and HER2 status, time to surgery, staging, chemotherapy regimen, localization method, pathology, and adjuvant therapy were analyzed. Pathologic complete response (pCR) of the CLN was observed in 41 patients (50.6%): 18.8% HR+/HER2−, 75% HR+/HER2+, 75% HR−/HER2+, and 62.5% triple-negative breast cancer (*p*-value = 0.006). CLN = SLN in 68 (84%) patients, while CLN = non-SLN in 13 (16%). In 14 (17.3%) patients, the final treatment was altered based on +CLN status: 11 patients underwent axillary lymph node dissection (ALND), and 3 had systemic treatment changes. pCR rates varied, with the highest conversion rates observed in HER2+ disease and the lowest in HR+/HER2− disease. In 2 (2.5%) patients, adjuvant therapy changes were made based on a non-sentinel CLN, while in 97.5% of patients, a SLN biopsy alone represented the status of the axilla. This demonstrates that a +CLN often alters final plans and that, despite also being a SLN in most cases, a subset of patients will be undertreated by SLN biopsy alone.

## 1. Introduction

Axillary lymph node status in breast cancer guides locoregional and systemic treatment decisions and continues to be an important prognostic factor. In patients with positive axillary nodes, the use of neoadjuvant chemotherapy (NAC, systemic therapy given prior to surgical intervention) can potentially downstage the axilla, with resolution of nodal metastasis ranging from 40% to 75%, depending on the underlying tumor phenotype and systemic therapy regimen [1,2,3]. NAC also provides an opportunity to assess the response of systemic therapy to further guide subsequent treatment decisions, including the use of radiotherapy and adjuvant therapies. Traditionally, in patients with documented axillary nodal disease prior to NAC, surgery has entailed a complete axillary lymph node dissection (ALND). The morbidity associated with ALND is significant and can include short-term and long-term sequelae including seromas, paresthesia, and lymphedema [4,5].

To harness the benefit of NAC in downstaging disease and the potential to de-escalate axillary surgery, several prospective trials were designed to determine if sentinel lymph node (SLN) biopsy alone could accurately assess response to pre-operative therapy in order to identify those who could be spared an ALND [6,7,8]. However, in these trials, the false negative rates (FNR) exceeded the prespecified targets (ranging from 12.6% to 14.2%), with authors concluding that the limitations of SLN after NAC precluded its use as an alternative to ALND unless additional techniques to improve sensitivity were employed. Methods cited to lower the FNR included the use of dual tracers, as well as increasing the number of SLN retrieved. In an important post-hoc analysis of one of the trials, Boughey and colleagues noted that when a clip was placed into a positive node prior to the initiation of NAC, with subsequent removal of the clipped node during the SLN surgery, the FNR was reduced [9].

Targeted axillary dissection (TAD) describes this intentional removal of a previously localized positive lymph node or clipped lymph node (CLN) and concurrent lymphatic mapping with a SLN dissection, performed to improve the pathologic evaluation of lymph nodes and decrease the false negative rate of SLN biopsy after NAC [10]. One of the initial prospective studies demonstrated an FNR as low as 2.0% with the addition of the clipped node to the routine SLN dissection [10]. Several studies have evaluated various aspects of selective removal of nodes biopsied prior to NAC including feasibility, choice of localization method, and ability to assess pathologic response to systemic preoperative chemotherapy and targeted therapies based on tumor receptors [10,11,12,13]. Should no residual disease be identified with this targeted axillary evaluation, then complete axillary dissection is omitted.

Our study aimed to examine patients with clinically node-positive (cN+) disease undergoing NAC and CLN localization to determine the rate of CLN being a non-SLN, identify factors associated with pathologic complete response (pCR, the absence of residual invasive cancer in tissue removed during surgery after treatment with neoadjuvant chemotherapy), and assess whether the status of the CLN impacted treatment recommendations.

## 2. Materials and Methods

Patients with biopsy-proven clinically node-positive disease (cN+), confirmed by either needle biopsy or fine needle aspiration and receiving NAC, were entered into a prospectively maintained, Health Insurance Portability and Account Act (HIPAA)-compliant, Institutional Review Board (IRB)-approved (University of South Florida IRB [MCC#16026, approval date 31 July 2009, last modification approval date 14 September 2023; MCC#20569, approval date 31 July 2019]) database. Informed consent was waived for this de-identified retrospective review of existing data. We identified patients treated between 2016 and 2022 who clinically converted to cN0 by physical exam and/or imaging and underwent TAD. All patients had preoperative localization of a single CLN performed at Moffitt Cancer Center and underwent dual tracer lymphatic mapping with isosulfan blue dye and technetium-99m sulfur colloid at the time of surgery, with confirmation of removal of the clipped node by intraoperative specimen radiograph.

Demographics, Hormone Receptor status (positive: ER and/or PR positive; negative: both ER/PR negative), HER2 receptor status, time from diagnosis to surgery, clinical and pathologic staging and node status, chemotherapy regimen, localization method, surgical pathology, treatment plan alterations based on CLN (cases where a + CLN with negative SLN led to completion axillary lymph node dissection or adjuvant chemotherapy), and adjuvant therapy were analyzed. The Kruskal–Wallis test, Chi-square test, or Fisher’s exact test, if applicable, were applied. The primary endpoint of the study was to determine the rate of the CLN being a non-SLN and its impact on therapeutic recommendations. The secondary endpoint was to evaluate factors associated with pCR.

All statistical tests were two-sided, with a significance level set at *p* < 0.05. The analyses were conducted using SAS (Version 9.4, SAS Institute Inc., Cary, NC, USA).

## 3. Results

### 3.1. Clipped Lymph Node

We identified 81 patients with a median age of 53.5 years (range, 25–79 years). The majority of cancers (98.8%) were invasive ductal carcinomas: 16 (19.8%) were HR+/HER2− tumors, 8 (9.9%) were HR−/HER2+, and 16 (19.8%) were triple-positive and triple-negative, respectively. At presentation, 8 patients (9.9%) had T1 tumors, 52 (64.2%) had T2 tumors, and 19 (23.5%) had T3 tumors; 76 (93.8%) of patients had clinical N1 disease at presentation, with 5 (6.2%) having cN2/N3 disease (Table 1).

A total of *70/81 (86.4%)* patients received post-NAC imaging to confirm response prior to definitive surgery. Clipped lymph nodes were primarily localized using post-chemotherapy preoperative radar reflector localization (73 cases, 90.1%). 80 patients (98.8%) were able to undergo TAD during surgery, with one patient requiring ALND due to significant fibrosis and lack of mapping/ability to detect CLN or SLN. A total of 79 patients (97.5%) had both CLN and SLN identified at the time of surgery. The median number of TAD nodes obtained per patient was 4 (range 1–10), with a median number of pathologic positive nodes of 1 (range 0–8) (Table 2).

A pCR of the CLN (cN+ to pN0 conversion) was noted in 41 patients (50.6%) with the following tumor markers: 18.8% of HR+/HER2−, 75% of HR+/HER2+, 75% of HR−/HER2+, and 62.5% of triple-negative (*p*-value = 0.006) (Table 3).

The CLN was the SLN in 68 (84%) patients, while the CLN was a non-SLN in 13 (16%) of the cases. The CLN had no impact on the final treatment plan in 67 (82.7%) patients compared to 14 (17.3%) cases, in which the final treatment plan was altered based on CLN status: with 11 patients undergoing ALND and 3 having changes to systemic treatment. However, for the majority of these patients, these changes in treatment plan were not solely dependent on the CLN status: either the CLN was also a SLN or in the cases in which the CLN was not the SLN, the TAD results (CLN + SLN) indicated the status of the axilla by SLN alone. A total of 2 cases (2.5%) had alteration of the final treatment plan based on positive non-sentinel CLNs. A total of 20 patients underwent completion ALND for residual nodal disease, with 6 patients (30%) having additional nodal disease identified at axillary dissection (Table 2). Of these patients who underwent completion ALND for residual nodal disease, 3 were HR+/HER2−, 2 were TNBC, and 1 was HR+/HER2+.

Postoperatively, 26 (32.1%) patients received additional chemotherapy, 40 (49.4%) received endocrine therapy, 9 (11.1%) received both adjuvant chemotherapy and endocrine therapy, and 80 (98.8%) patients received radiation therapy.

### 3.2. Factors Associated with Patholgic Complete Response (pCR)

In univariate association analysis, clinical tumor size was significantly associated with the conversion of CLN+ to pN0 (*p* = 0.041). Also, the tumor subtype was significantly associated with CLN pCR (*p* = 0.006). The highest rates of CLN pCR were seen in patients with HR+/HER2+ and HR−/HER2+ disease, both with pCR rates of 75%. Patients with triple-negative breast cancer also had high CLN pCR rates of 62.5%. The lowest response rate was in HR+/HER2− patients: the CLN pCR rate was 18.8%. Chemotherapy regimen was associated with pCR (*p* = 0.027), but the effect was primarily driven by dual HER2 blockade in HER2 positive patients. A pCR in the breast was also significantly associated with conversion of cN+ to pN0 (*p* < 0.001). Also associated with a pCR in the clipped node was a pCR in the SLN (*p* < 0.001). A pCR in breast was noted in 34 patients (42%). There were 5 patients who had a breast pCR but no pCR in the CLN (Table 3).

## 4. Discussion

De-escalation of axillary surgery is a primary focus of modern breast surgery, and NAC has introduced new possibilities for patients with a clinically positive axilla and subsequent pCR [8,9]. Over the last decade, improvements in systemic therapy regimens have drastically increased pCR rates, especially in patients with HER2 positive and triple-negative subtypes [1,3,13,14,15,16,17]. In our study, we observed varying nodal pCR rates based on tumor receptors, consistent with other studies. Given the current practice of dual HER2 blockade with trastuzumab and pertuzumab, our pCR rate for HR−/HER2+ and HR+/HER2+ were each 75%. Our nodal pCR rate falls in line with HER2 + nodal pCR in the literature ranging from 51.7% to 97% [1,3,13,16,17]. We also found a higher nodal pCR rate in patients with TNBC (62.5%) which is slightly higher than what has been reported in other studies, ranging from 40–50% in the literature [1,10,13,14,17]. The higher rate of pCR in this cohort of patients may be attributed to the fact that the majority of our cohort had cN1 disease (93.8%), reflecting an overall lower burden of disease which may correspond to improved response. In HR+/HER2− patients we saw an 18.8% nodal pCR rate, which falls in range with other studies showing a pCR rate varying between 11–33% [13,16,17,18].

With NAC regimens improving the response rates in axillary disease, the hope is to avoid complete axillary dissection for those who respond well to treatment. The initial purpose of lymphatic mapping with SLN biopsy was for staging, to inform therapeutic options including systemic therapy, radiation, and the potential need for ALND. In patients with clinically node-positive disease who undergo NAC, lymphatic mapping with SLN biopsy is employed to assess response to chemotherapy with recognized limitations [9,19,20,21]. For those who do not have a nodal pCR after initially presenting with cN+ disease, it remains important to identify those patients for additional adjuvant therapies, such as further surgery, radiation or additional adjuvant systemic therapy such as capecitabine for residual triple-negative disease as described in the CREATE-X trial [22] or trastuzumab emtansine for residual HER2 disease as described in the KATHERINE Trial [23].

The necessity of removing the clipped node at the time of a SLN biopsy (TAD) versus SLN biopsy alone is controversial, with some literature suggesting that SLN biopsy alone is adequate after NAC if 3 or more nodes are removed at the time of surgery [13,24,25,26]. In our study, we found that the CLN was simultaneously a SLN in 84% of cases, but in the 16% cases in which the CLN was not a SLN, the majority of these cases did not subsequently have changes in treatment; this was due to the fact that either the status of both the CLN and SLN were the same (either both had a pCR or both still remained positive) or there was residual disease in the breast driving the change in treatment. In the cases in which the CLN remained positive, the final treatment plan was changed in 14 (17.3%) cases, with the main impact on treatment plan being a completion ALND. Of note, only 2 of these 14 patients with treatment changes based on the CLN (2.5% of total cohort) had a CLN which was a non-SLN and the treatment changes were based solely on the status of the CLN: one underwent an ALND for residual disease in the CLN with a pCR in the SLN and another avoiding an ALND because the CLN had a pCR but there was no SLN due to failed lymphatic mapping. Consistent with other studies, the pCR rate is markedly lower among HR+/HER2− patients, suggesting that TAD is, perhaps, a more suitable operation for triple-negative or HER2+ disease [1]. In our cohort, 50% of the patients who had additional positive nodes upon completion ALND had HR+/HER2− disease, with the number of positive residual nodes also higher in these patients. However, it must be noted that within our cohort, some patients did not proceed with completion ALND due to patient preference, participation in ongoing clinical trials, or multidisciplinary team discussion, which could have affected our results with such a small sample size.

In our small retrospective cohort, in 97.5% of the patients, a SLN biopsy alone represented the status of the axilla. Thus, the overwhelming majority of these patients would not have had changes in therapy despite the omission of TAD. Longitudinal studies with a larger sample size will be paramount for assessing those patients where the CLN was not a SLN and who had therapy changes, i.e., those who would have been wrongly classified as an axillary pCR, thereby risking undertreatment in the adjuvant setting. Additionally, it should be investigated whether this subset of patients remains at 2.5% with a larger sample size. Recent studies have demonstrated performing SLN biopsy without a simultaneous TAD in CN+ patients undergoing NAC does not appear, with limited long-term data, to have impacted axillary recurrence or survival rate [24,25,26], but the question remains if this specific cohort of patients who have been undertreated have a significantly different axillary recurrence rate or overall survival difference. Further studies looking at these patients should help shed light on this area. It may also be beneficial to integrate newer axillary imaging technology and techniques into future studies, such as shear wave elastography and deep learning radiomics of ultrasonography [27,28,29]. These imaging adjuncts may prove helpful in predicting axillary pCR after neoadjuvant therapy has been completed and help guide patient selection and operative technique prior to surgical evaluation of the axilla.

Furthermore, it is possible that undertreated patients could be at a higher risk of recurrence in the future as we also aim to de-escalate regional nodal radiotherapy (RNRT). Current randomized controlled trials such as NRG Oncology/NSABP B-51/RTOG1304 aim to determine whether radiotherapy in cN+ that has a pCR after NAC can be de-escalated [30]. In our cohort, 98.8% of the patients underwent adjuvant radiotherapy, with 97.5% undergoing RNRT based on cN+ status, so a false negative pCR based on the axillary status could impact these patients. The Alliance A011202 trial [31,32] and ADARNAT trials aim to compare RNRT with and without completion ALND in patients with pN+ disease following NAC, and RNRT vs. completion ALND in patients with pN+ disease following NAC, respectively [32,33], which may also provide more insight into how de-escalation of RT might affect outcomes for our subset of undertreated patients.

There are multiple limitations to this study. First, this is a single-institution retrospective non-randomized study with no control group for comparison, so there are likely confounding variables that affect our study results. Not all patients who had residual axillary disease went on to receive a completion ALND either at the discretion of a multidisciplinary team due to low volume residual disease or the number of TAD nodes removed, enrollment in the Alliance A011202 trial, or patient refusal. It is also possible that our study sample may have been underpowered to account for more subtle differences regarding the rate of the clipped node not being a sentinel node and possibly impacting therapy changes. Additionally, a selection bias could be present by initially choosing patients with an overall low nodal burden to perform a TAD, as patients with a higher burden of disease may have proceeded straight to axillary dissection.

## 5. Conclusions

In this NAC population, there was a significant difference in pCR rates, with the highest conversion rates in HER2-positive disease and the lowest in HR+/HER2− disease. The CLN was a non-SLN in 16% of the patients, and a +CLN impacted the final treatment plan in almost 17% of the cases, but in only 2.5% of the cases was the targeted dissection necessary to see changes in adjuvant management. Our data demonstrates that although a +CLN altered final treatment plans and, in some cases, represented patients who would have been undertreated by SLN biopsy without a TAD, in the overwhelming majority of patients, the SLN biopsy alone gave us the appropriate status of the axilla. As with other studies, we noted that pCR rates are the lowest in HR+/HER2− disease, and this cohort of patients was also more likely to have a higher residual nodal burden on completion ALND, suggesting a possible benefit to selectively performing TAD on these patients. Longitudinal follow-up for evidence of recurrence, as well as a larger cohort and prospective studies, are needed to enhance our understanding of this topic to ultimately help better predict pCR rates, stage the axilla, select patients most likely to benefit from a TAD, and guide adjuvant therapy while de-escalating axillary morbidity in our patients.

## Figures and Tables

**Table 1:** Clinicopathologic characteristics.

	Median (Range)
Age at diagnosis	53.5 (25–79)
	N (%)
Clinical T stage	
T1	8 (9.9)
T2	52 (64.2)
T3	19 (23.5)
Unknown	2 (2.5)
Histology	
Ductal	80 (98.8)
Lobular	1 (1.2)
Tumor subtype	
HR+/HER2+	16 (19.8)
HR+/HER2−	16 (19.8)
HR−/HER2+	8 (9.9)
TNBC	16 (19.8)
Other	25 (30.9)
Clinical node status	
N1	76 (93.8)
N2/N3	5 (6.2)

**Table 2:** Lymph node status.

	Median (Range)
Number Nodes Removed (CLN + SLN or ALND)	5 (1–27)
Number SLN	4 (1–10)
Number SLN positive	1(0–8)
Number Positive Nodes	1 (0–11)
	N (%)
Rate of Localized Node (CLN = SLN)	
Yes	68 (84.0)
No	13 (16.0)
CN+ to pN0 Conversion	
Yes	41 (50.6)
No	40 (49.4)
Axillary Node Dissection	
Yes	20 (24.7)
No	61 (75.3)

**Table 3:** Univariate association with cN+ to pN0 conversion.

		CN+ to pN0 Conversion		
Covariate		No	Yes	*p*-Value
		N = 40	N = 41	
Clinical Tumor Size	T1	4 (50)	4 (50)	0.041
	T2	31 (59.6)	21 (40.4)	
	T3	5 (26.3)	14 (73.7)	
Chemotherapy Regimen	ACT	30 (61.2)	19 (38.8)	0.027
	TCHP	7 (28)	18 (72)	
	Other	3 (42.9)	4 (57.1)	
pCR Breast	No	35 (74.5)	12 (25.5)	<0.001
	Yes	5 (14.7)	29 (85.3)	
pCR SLN	No	34 (97.1)	1 (2.9)	<0.001
	Yes	3 (9.7)	28 (90.3)	
	Unknown	3 (20)	12 (80)	
Tumor Subtype	HR+/HER2+	4 (25.0)	12 (75.0)	0.006
	HR+/HER2−	13 (81.3)	3 (18.8)	
	HR−/HER2+	2 (25.0)	6 (75.0)	
	TNBC	6 (37.5)	10 (62.5)	
	Other	15 (60.0)	10 (40.0)	

## Data Availability

The datasets presented in this article are not readily available due to privacy and ethical restrictions. Requests to access the datasets should be directed to the corresponding authors.
